# Psychosocial Challenges Facing Young People With Inherited Metabolic Disorders and Their Parents: A Systematic Review

**DOI:** 10.1002/jmd2.70000

**Published:** 2025-02-25

**Authors:** Clara Sherlock, Kim Clarke, Norah Jordan

**Affiliations:** ^1^ National Centre for Inherited Metabolic Disorders, Children's Health Dublin Ireland

## Abstract

Recent advancements in new‐born screening have reduced the risk of life‐threatening complications associated with inherited metabolic disorders. However, the risk of negative psychosocial effects on families persists. The aim of the present study was to systematically review the literature concerning the psychosocial challenges experienced by young people with metabolic conditions and their families, to inform the development of supports that meet the needs of those linked with metabolic services. The electronic databases MEDLINE, CINAHL, PsychInfo, and Psychology and Behavioural Sciences Collection were searched for studies examining psychosocial challenges reported by families with inherited metabolic conditions, over the last two decades. Five‐thousand sixty‐seven articles were screened for relevance. Twenty‐nine studies met the inclusion criteria. Study quality and reliability were independently assessed by two reviewers. Results highlighted the myriad of physical, social, psychological and practical challenges experienced by young people with metabolic conditions and their families. These challenges included social isolation, burden of care, and learning and emotional difficulties. Findings reiterate the importance of developing peer support groups and delivering psychoeducation to families, as well as the central role psychology and social work should play in metabolic MDTs, to improve families' experiences and outcomes.

**Summary**

- The present review is the first to examine the psychosocial impact of IMDs on young people with IMDs and their families.
- All of the studies included in this review identified significant practical, social, psychological and/or physical challenges with managing a life‐long condition.
- This review provides insights that may be utilised by healthcare providers to develop supports that meet the psychosocial needs common to children with IMDs and their families.

## 1 Introduction

Inherited metabolic disorders (IMDs) represent over 1400 heterogeneous genetic diseases [1]. IMDs stem from inborn errors of metabolism, most commonly involving enzyme or transporter deficiencies that impact expected metabolic processes [2]. Though individually rare, their overall incidence of approximately 1 in 1000 live births indicates that IMDs are collectively common [3]. While advances in new‐born screening have reduced the risk of life‐threatening complications developing for many with IMDs [4], the risk of adverse long‐term outcomes remains [5]. Abnormal IMD‐related molecule function can disrupt neurodevelopment at any stage, giving rise to an array of potential clinical presentations [6]. Accumulations of toxic metabolites can lead to irreversible neurological damage, manifesting as progressive declines in cognitive function or developmental regression [7, 8]. Such accumulations can also have significant physical impacts such as increased pain, fatigue [9], organ failure and cardiovascular issues [10]. Those with IMDs can equally present with movement disorders such as ataxia, dystonia and choreoathetosis [10] and in some cases cerebral palsy [11]. However, the impacts of IMDs are not solely medical or neurological in nature.

A number of psychosocial stressors are inherent in living with a chronic condition. Many caregivers struggle with the burden of multiple hospital visits, managing medications and uncertainty about the future [12]. As many chronic diseases develop from infancy and progress throughout adolescence, IMDs pose additional challenges that can affect the entire family system [13]. IMD diagnoses can often require extensive clinical and laboratory investigation. Medical interventions can equally necessitate invasive procedures, repeated blood sampling, specialised diets and crisis management [14]. Parents often experience condition‐specific challenges that affect the well‐being of the entire family unit [15]. Parents of children with intoxication‐type disorders have reported that dietary requirements imposed both social and emotional burdens, as their own limited understanding of IMDs rendered it difficult to communicate their needs to others [16]. Parents of children with metabolic disorders have also reported increased anxiety and depression, with all caregivers reporting significant stress due to difficulty navigating social environments [17]. Few studies have explored the effect of IMDs on young people with the diagnosis, though the negative impacts on their quality of life have been highlighted [18].

A detailed understanding of the psychosocial challenges faced by those with IMDs is needed to develop targeted treatments that improve outcomes for young people and their families. However, the comparative lack of research concerning the psychosocial impacts of IMDs calls into question the extent to which current treatments for patients and their families are best suited to meeting psychosocial needs [13]. Although a recent systematic review examined the psychosocial impact of parenting a child with a lysosomal storage disorder [19], the overall psychosocial impacts of IMDs on parents and children are yet to be reviewed. Considering that parent and child perspectives may differ, the dearth of research on young populations highlights a significant gap in our understanding of the specific psychosocial challenges faced by young people with IMDs and how these may impact wider family function [17]. Given that many centres that treat IMDs function as a single unit, it is useful to consider overall psychosocial needs, as interventions are likely to be delivered in this way.

Though individually rare, it is expected that all healthcare professionals will encounter a number of children with IMDs throughout their career [2]. An understanding of the overall impact of IMDs is key to providing treatment and care that optimises families' quality of life and minimises difficulties or trauma related to ongoing, significant or prolonged distress [15]. Therefore, the aim of the present study was to systematically review the literature concerning the psychosocial challenges faced by young people with IMDs and their families. Experiences common to all children with IMDs and their families were collated to assist healthcare professionals in developing effective trans‐diagnostic supports that meet the needs of many families attending IMD services.

## 2 Methods

The reporting of data in this review was informed by the PRISMA 2020 statement [20]. The review protocol was registered on PROSPERO and can be accessed using the ID: CRD42023413936. The population for the present review was young people (under the age of 18 years) with IMDs, parent(s) or carer(s) of young people with IMDs, and/or siblings of young people with IMDs. The intervention was usual medical treatment for the young person's IMD. Comparison groups consisted of young people without IMDs or young people with differing IMD diagnoses. Outcomes related to quality of life and psychosocial function. Study types were quantitative, qualitative or mixed‐methods.

### 2.1 Search Strategy

The following electronic databases were searched on 6 April 2024: MEDLINE, CINAHL, PsychInfo and Psychology and Behavioural Sciences Collection. Reference lists of included studies were manually searched for relevance, ensuring research saturation. The following search terms were used in searches: (1) (inborn errors of metabolism OR inherited metabolic dis* OR metabolic dis OR familial hypercholesterolaemia OR galactosemia OR galactosaemia OR glutaric aciduria type one OR GA1 OR glycogen storage disorder OR GSD OR Homocystinuria OR HCU OR LARS OR Infantile Liver Failure Syndrome type IOR LCHADD OR long chain 3‐hydroxyacyl‐CoA dehydrogenase deficiency OR lysosomal storage disorder* OR LSD OR fabry disease OR hurler syndrome OR mucopolysaccharidosis type one OR I‐cell disease OR mucolipidosis type II OR maple syrup urine disease OR MSUD OR MCADD OR medium chain acyl‐CoA dehydrogenase deficiency Or Mitochondrial Disorder OR Methylmalonic Acidaemia OR MMA OR Phenylketonuria OR PKU OR propionic acidaemia OR PA OR tyrosinaemia type one OR urea cycle disorder* OR VLCADD OR very long chain acyl‐CoA dehydrogenase deficiency); (2) (famil* OR parent* OR mother OR father OR sibling OR child* OR adolescen* OR teenager OR young person); and (3) (challenges OR difficulties OR adherence OR acceptance OR adjustment OR quality of life OR needs). The year of publication was limited to the last two decades. No other filter was applied to searches.

### 2.2 Study Selection

The systemic review software, Covidence, was used for article access, screening, quality appraisal and data extraction (Veritas Health Innovation, Melbourne, Australia. Available at www.covidence.org). This software also facilitated the removal of duplicate articles. Two independent researchers (C.S. and K.C.) initially screened articles for suitability by titles and abstracts. Full‐text screening was then carried out by the same researchers with disagreements resolved through discussion. The inclusion criterion was studies measuring at least one psychosocial outcome of young people with IMDs and/or their caregivers. All IMDs, ethnicities and nationalities were included. Exclusion criteria were (1) studies including adults with IMDs, (2) review papers and (3) papers unavailable in English.

### 2.3 Data Extraction and Quality Appraisal

Articles meeting inclusion criteria were appraised by two independent researchers (C.S. and K.C.) using the Joanna Briggs Institute (JBI) critical appraisal tools for quantitative and qualitative studies [21]. These tools assess the trustworthiness, relevance and results of articles. Data were extracted by the same researchers using a bespoke data extraction form. Data were synthesised by transforming quantitative findings into qualitative findings using data‐based convergent synthesis [22].

## 3 Results

### 3.1 Literature Search

**FIGURE 1:**
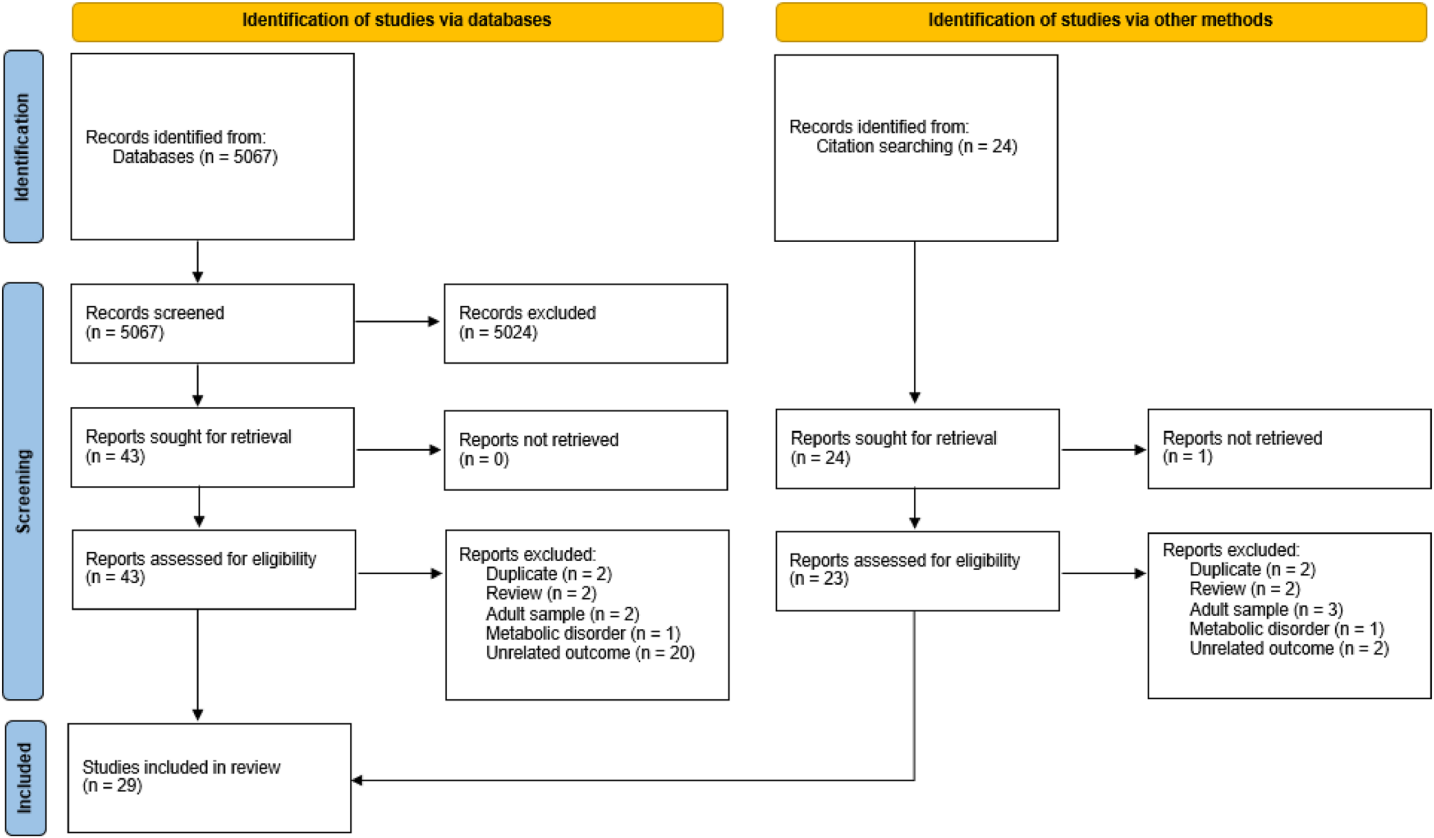
PRISMA 2020 diagram.

The search yielded 5067 papers whose titles and abstracts were screened for relevance. Forty‐three papers were sought for full‐text review. Sixteen of these studies met the inclusion criteria for this review. A further 23 articles were sought for full‐text review following an ancestry search of the included articles. Thirteen of these studies met the inclusion criteria. Twenty‐nine articles in total were included in this review (see Figure 1 for the PRISMA flow diagram).

### 3.2 Study Design

Twenty‐four studies adopted a quantitative design. All quantitative studies were cross‐sectional in nature. Here, psychosocial challenges were examined using a variety of standardised (*n* = 22) and bespoke (*n* = 5) measures. Challenges were primarily derived from measures of quality of life (*n* = 15), adaptive function (*n* = 3), child behaviour (*n* = 4) and stress (*n* = 6). Included measures are detailed in Table S1. The remaining five studies adopted a qualitative design. Qualitative studies explored the experiences of young people with IMDs and their parents through open‐ended interview (*n* = 1), semistructured interviews (*n* = 2) or focus groups (*n* = 2). Research methodologies were primarily phenomenological approaches though one study used grounded theory.

### 3.3 Study Quality

When critical appraisal of studies was concerned, many of the included studies were awarded either a perfect (*n* = 6) or near perfect (*n* = 4) score. The majority of studies failed to account for and/or describe the strategies used to cope with confounding variables such as the presence of comorbidities (*n* = 11) in quantitative studies and failed to describe the theoretical underpinning of qualitative studies (*n* = 5), resulting in lower appraisal scores. However, no study was awarded fewer than four points (out of seven). All quantitative studies clearly identified inclusion and exclusion criteria and described the study participants and setting in appropriate detail. All but two studies [23, 24] measured outcomes using standardised assessment tools, and all studies used appropriate statistical analysis. Only one qualitative study failed to demonstrate congruity between their stated philosophical perspective, research methodology and research objectives [25]. Though congruity was established between research methodology as analysis of data in this study, as well as the authors' interpretation of results. Participant voices were adequately represented in all included qualitative studies and conclusions drawn by authors were clearly derived from their analysis of the data.

### 3.4 Participant Characteristics

Most studies were diagnosis‐specific, focusing on phenylketonuria (PKU; *n* = 16), mucopolysaccharidosis type II (MPS II; *n* = 3), tyrosinemia type I (TT1; *n* = 1), succinic semialdehyde dehydrogenase deficiency (SSDD; *n* = 1), methylmalonic academia (MMA; *n* = 1) and glycogen storage disease (GSD; *n* = 1). Six studies examined multiple metabolic conditions. Sample size in these studies ranged from 5 to 320 participants. Fourteen studies included samples of parents only, six studies included parents and their children, and eight studies included only young people. Parents' age ranged from 34 to 43 years. Young people's age ranged from 2 to 20 years. Two studies included only mothers [26, 27]. Only one study included siblings [28]. Demographic characteristics of included samples are outlined in Table 1.

**TABLE 1:** Demographic characteristics of included studies.

First author, year	Study design	Country	Sample	Child's metabolic condition	*N*	Age* mean standard deviation	Gender male female
Bose, 2021 [29]	Qualitative	USA	Parents	SSDD	5	n/r	M: 1 F: 4
Arpaci, 2020 [26]	Cross‐sectional	Turkey	Mothers	GSD PKU HFI FH MSD MSU MA G GD1 Other	47 9 7 6 7 3 4 2 2 2 2	M: 36.93 SD: 5.22	F: 47
Morawska, 2020 [27]	Cross‐sectional	Australia	Mothers	PKU	18	M: 38.67 SD: 5.96	F: 18
Ambler, 2018 [35]	Cross‐sectional	Ireland New Zealand UK	Parents Control parents	PKU	38 32	M: 40.08 SD: 5.36 M: 38.0 SD: 6.12	M: 6 F: 32 M: 8 F: 24
Irannejad, 2018 [43]	Cross‐sectional	Iran	Parents	PKU	124	M: 39.63 SD: 10.62	M: 62 F: 62
Yamaguchi, 2018 [28]	Cross‐sectional	Japan	Adolescents Siblings Primary caregiver Caregiver partner	AAD LSD OA DACM MMD FAMD	56 31 6 9 4 4 2 35 143 86	M: 12.0 SD: 3.1 M: 12.4 SD: 3.3 M: 42.4 SD: 6.0 M: 43.3 SD: 6.8	M: 28 F: 28 M: 11 F: 22 N/R: 2 M: 11 F: 132 M: 76 F: 10
Campbell, 2018 [30]	Cross‐sectional	USA	(Grand) parents	TT1	26	M: 36.44 SD: 7.19	M: 5 F: 20 N/R: 1
Carpenter, 2017 [40]	Qualitative	UK	Parents	PKU	7	n/r	M: 1 F: 6
Medford, 2017 [45]	Cross‐sectional	UK	(Grand) parents	PKU	46	M: 36.92 SD: 8.83	M: 45 F: 1
Splinter, 2016 [38]	Cross‐sectional	USA	Caregivers	MA	35	n/r	n/r
Witalis, 2016 [24]	Cross‐sectional	Poland	Young people Parents	PKU	218 178	173 ≥ 10–19 years 45 > 19 years n/r	M: 110 F: 108 M: 46 F: 132
Gunduz, 2015 [42]	Cross‐sectional	Turkey	Parents Healthy Controls	PKU	61 36	M: 34.3 SD: 5.8 M: 35.8 SD: 6.7	M: 18 F: 43 M: 7 F: 29
Needham, 2015 [46]	Cross‐sectional	Australia Canada Ireland UK USA	Adolescents Parents	MPS II	21 73	M: 12.52 SD: 8.88 n/r	M: 21
Needham, 2014 [46]	Cross‐sectional	Australia Canada Ireland UK USA	Adolescents Parents	MPS II	21 73	M: 12.52 SD: 8.88 n/r	M: 21
Eminoglu, 2013 [50]	Cross‐sectional	Turkey	Adolescents	OA DAAM DACM	68 14 21 33	n/r	M: 35 F: 33
Fabre, 2013 [32]	Cross‐sectional	France	Children Parents	OA UCD MSUD	21 10 6 5 21	M: 8.25 SD: 5.6 M: 39.14 SD: 6.64	M: 10 F: 11 M: 10 F: 11
Fidika, 2013 [41]	Cross‐sectional	Germany	Parents	PKU	89	M: 39.0 SD: 6.9	M: 12 F: 76
Raluy‐Callado, 2013 [23]	Cross‐sectional	Brazil Germany UK UKA	Adolescents Parents	MPS II	96 96	M: 14.2 SD: 6.7 M: 42	M: 96 M: 14 F: 82
Sharman, 2013 [37]	Qualitative	Australia	Adolescents	PKU	8	M: 14.25 SD: 2.18	M: 3 F: 5
Di Ciommo, 2012 [25]	Qualitative	Italy	Adolescents	PKU	20	6 < 10 years 14 > 10 years	M: 12 F: 8
Cotugno, 2011 [31]	Cross‐sectional	Italy	Adolescents	PKU	41	M: 10.58 SD: 6.0	M: 25 F: 16
Ten Hoedt, 2011 [47]	Cross‐sectional	The Netherlands	Parents	PKU G LSD OD MD Healthy	86 41 108 441	M: 40.7 SD: 6.3 M: 42.5 SD: 6.5 M: 41.5 SD: 7.0 M: 43.7 SD: 5.4	M: 50 F: 66 M: 27 F: 42 M: 23 F: 85 M: 74 F: 36
Wu, 2011 [49]	Cross‐sectional	China	Children Healthy controls	PKU	28 96	M: 1.89 SD: 0.8 n/r	M: 13 F: 15 n/r
Vegni, 2010 [39]	Qualitative	Italy	Adolescents	PKU	37	Range: 8–31	M: 20 F: 17
Hatzmann, 2009 [9]	Cross‐sectional	The Netherlands	Adolescents Parents	LSD OA MD	108 34 18 56 108	M: 8.7 SD: 4.4 M: 41.5 SD: 7.0	M: 54 F: 54 M: 23 F: 85
Lord, 2008 [44]	Cross‐sectional	Australia	Children Parents	PKU	55 99	M: 6.6 SD: 3.2 M: 35.2 SD: 5.2	M: 28 F: 27 M: 47 F: 52
Storch, 2008 [34]	Cross‐sectional	USA	Adolescents Healthy controls	GSD	31 42	M: 11.11 SD: 4.18 n/r	M: 16 F: 15 M: 21 F: 21
Van Zutphen, 2007 [48]	Cross‐sectional	USA	Children	PKU	15	M: 13.8 Range: 8–20	M: 7 F: 8
Bilginsoy, 2005 [36]	Cross‐sectional	USA	Parents	PKU	32	n/r	n/r

Abbreviations: DAAM, disorder of amino acid metabolism; DACM, disorder of amino carbohydrate metabolism; FAMD, fatty acid metabolism disorders; FH, familial hyperlipidaemia; G, galactosaemia; GD1, gaucher disease type 1; GSD, glycogen storage disease; HFI, hereditary fructose intolerance; MA, methylmalonic acidemia; MD, mitochondrial disorders; MMD, metal metabolism disorders; MPS II, mucopolysaccharidosis type II; MSUD, maple syrup urine disease; n/r, not reported; OA, organic acidemia; OD, organic disorders; PKU, phenylketonuria; SSDD, succinic semialdehyde dehydrogenase deficiency; TT1, tyrosinemia type 1; UCD, urea cycle defect.

* Age: Reported in years.

### 3.5 Challenges Facing Families

#### 3.5.1 Physical Challenges

Ten studies highlighted physical challenges associated with IMDs [9, 23, 26, 28, 29, 30, 31, 32, 33, 34]. Four of these studies included only parents [26, 29, 30, 32], two included only patients [31, 34] and four included both groups [9, 23, 28, 33]. Caregivers in two studies noted their children experienced fatigue, headaches, stomach aches, nausea and sleep difficulties [29, 30]. Muscle weakness, pain, diarrhoea and vomiting were also outlined by mothers in one study [26]. Physical challenges were self‐reported by young people, reflected in lower physical quality of life [28, 31, 34], physical function [23] and lower overall adaptive behaviour scores than norms [29]. These scores mirrored parent proxies [34]. Parents of children with IMDs equally self‐reported impoverished physical quality‐of‐life scores [32, 33], in addition to sleep impairment [9]. See Tables 1 and 2 for a summary of quantitative and qualitative findings.

**TABLE 2:** Summary of qualitative findings.

Challenges	Findings	First author, year
Physical		
Symptoms	Problems related to motor coordination, sleep patterns and fatigue, toilet training and walking	Bose, 2021 [29]
	The most common symptoms were muscle weakness (14.9%), bone pain (12.8%), diarrhoea (12.8%) and vomiting (12.8%), delayed growth (23.4%)	Arpaci, 2020 [26]
	Symptoms with the greatest impact were tiredness, impacting > 33% of subjects' children, stomach aches (15%), porphyria‐like symptoms (12%), insomnia (8%), nausea (8%), eye sensitivity (8%) and headaches (4%)	Campbell, 2018 [30]
Social		
Isolation	Overall decreased or generally negative interactions with peers. Inappropriate behaviours from patients; peers mimicking, mocking, excluding. Overall lack of independence of their child as a limiting factor to social interactions. Caregivers are isolated from friends and family members due to their responsibilities	Bose, 2021 [29]
	Recurring concepts included social isolation, strains on family relationships, a generally negative impact on the parents and child	Splinter, 2016 [38]
	Some reported poor psychological health, no social life and no support system	Needham, 2014 [33]
	Some young people avoid eating with others and sacrifice a part of their social life to hide their diagnosis	Vegni, 2010 [39]
	Odds of impairment of daily activities increased when a child had difficulties making contact and when a hobby was given up due to the child's illness	Hatzmann, 2009 [9]
	Problems with child's interaction with peers and the planning of family events such as eating out or holidays. Two major sources of stress related to food planning/preparation and ramifications of the diet for social life	Bilginsoy, 2005 [36]
Psychological		
Behaviour	Three young people described frequent defiance and opposition to authority, often related to their overall mood	Bose, 2021 [29]
	The most common psychosocial problems were anger and aggression (23.4%)	Arpaci, 2020 [26]
	Parents of children with an IMD reported more child behaviour problems than controls	Ambler, 2018 [35]
Difficult feelings	The most common psychosocial problems were anger and aggression (23.4%), developmental delay (27.7%) and anorexia nervosa (25.5%)	Arpaci, 2020 [26]
	The highest mean scores were for parental guilt related to poor adherence to dietary restrictions and phe‐free amino acid supplementation. Child's anxiety during blood tests also indicated a major impact. The child's anxiety during medical procedures had a significant impact on parental stress	Morawska, 2020 [27]
	Parents reported moodiness (23%), anxiety (8%), sadness (8%) and aggressiveness (8%) in their children. 81% of caregivers reported feeling guilty if their child's diet was not followed. 77% reported guilt when their child was nonadherent with their specialised formula. 69% of caregivers felt their diet had a moderate to severe influence on their QOL. Some parents perceived their children to be sad and anxious	Campbell, 2018 [30]
	Common themes for parents included the effortful creation of a normal life. Fear of their child feeling different. Emotional and social consequences of controlling children. Fears of the consequences of non‐compliance. Increased parental responsibility	Carpenter, 2017 [40]
	12% of the children surveyed stated that they perceived themselves as inferior/different from their peers. 28% of parents believed that their children felt inferior to their peers, increasing with age. 71% of parents experienced anxiety about their children's future. 42% of patients were concerned about their future. 66% of children (50% of parents) believed that their IMD affected their education	Witalis, 2016 [24]

	Common responses were worry or fear for their child's pain, the future level of functioning, how the patient will cope with having an IMD and social isolation. Approximately 20% of caregivers expressed that their greatest worry is what would happen to their children if the caregiver died	Needham, 2014 [33]
	Young people reported a profound sense of sadness (e.g., when offered food by friends/family). A highly challenging feeling of ‘difference’, fear of discrimination and stigmatisation	Di Ciommo, 2012 [25]
	Young people were conscious of being considered different. Had to decide who was worthy of trusting with information. Some young people feel supported by the assistance of their parents. Others were frustrated by their parents' attempts to control their diet and therefore felt stimulated to transgress	Vegni, 2010 [39]
	Frustration of young people with repeatedly having to provide explanations for food intake and that condition is not curable. Embarrassment and discomfort in disclosing medical condition	Sharman, 2009 [37]
	33% of mothers indicated experiencing a high level of distress	Lord, 2008 [44]
	Depression and anxiety scores were significantly higher in young people with IMDs	VanZutphen, 2007 [48]
Parents expressed varying degrees of adjustment to living with an IMD, with responses ranging from ‘hell’ and ‘it's a mess’ to ‘positive’	Bilginsoy, 2005 [36]
Learning	Symptoms with the greatest impact were lack of concentration, slow thinking, and irritability, impacting > 33% of subjects' children	Campbell, 2018 [30]
	16% parents reported that their children had learning problems	Bilginsoy, 2005 [36]
Practical		
Burden of care	Considerable burden with the multitude of tasks required in being caregiver. Differences in family experiences and a perceived lack of understanding from others	Bose, 2021 [29]
	Mothers had the highest mean scores on the time‐dependence burden	Arpaci, 2020 [26]
	Greatest perceived stress with the question: ‘How many times did you find yourself thinking about the tasks that you have to do in the last month?’	Irannejad, 2018 [43]
	Difficulties in finding low‐protein foods, finding time to prepare different meals for different members of the family, record keeping	Bilginsoy, 2005 [36]
Finances	Recurring difficulties included financial burden	Splinter, 2016 [39]
	68.8% of families stated that it was a financial burden to have a supply of low‐protein products	Gunduz, 2015 [42]
Lack of knowledge	Common theme for parents were lack of knowledge, understanding and information around IMD	Carpenter, 2017 [40]
	Different IMD definitions lead to an uncertainty about diseases and possible consequences. Scarcity of information resulting in patients filling their own knowledge gaps	Vegni, 2010 [39]
	Young people finding it hard to interpret food labels. Problems in explaining their condition and dietary requirements to other people	Sharman, 2009 [37]
Planning	Practical impact of the dietary restriction	Morawska, 2020 [27]
	Difficulty with both restriction and unpalatable special food	Di Ciommo, 2012 [25]
	Difficulties in the preparation and storage of supplemental formula. Struggle to find appropriate foods when eating at restaurant, temptation of ‘forbidden’ foods. The primary factor identified as interfering with dietary adherence was social activities (i.e., eating out, going to camps, holidays)	Sharman, 2009 [37]
	Problems with the planning of family events such as eating out or holidays. Two major sources of stress related to food planning/preparation and ramifications of the diet for social life	Bilginsoy, 2005 [36]

#### 3.5.2 Social Challenges

Ten studies outlined the social challenges faced by families with IMDs [9, 29, 32, 33, 34, 35, 36, 37, 38, 39]. Five of these studies included only parents [29, 32, 35, 36, 38], three included only patients [34, 37, 39] and two included both groups [9, 33]. Parents in three studies specified isolation, experienced by themselves and/or their children, as a significant challenge [29, 33, 38]. Parents noted that their children had difficulty interacting with [36], and were excluded or mocked by, peers [29]. This social isolation was reflected in significantly low scores on parent proxy assessment of social functioning [34, 38] and social quality of life [32]. Parents also recognised that their child's lack of independence hindered their social interactions [29], as did being forced to give up hobbies due to illness [9]. Social challenges were equally highlighted by young people. One study explained how young people felt that social interactions were impacted by treatments such as dietary restrictions, as they struggled to explain their dietary requirements to others and to find restaurants that served appropriate foods [37]. Young people in one study described avoiding eating with others, sacrificing this part of their social life to hide their condition [39]. Parents also identified eating out as posing a social barrier for the entire family unit [36]. Parents themselves experienced isolation from family and friends due to their responsibilities as carers, in addition to a perceived lack of others' understanding of IMDs [29]. Parents noted having ‘no social life’ or support system [33] and highlighted the strain that illness had placed on familial relationships [38]. Parents also self‐reported lower scores than norms on the assessment of social support [35].

#### 3.5.3 Psychological Challenges

Twenty‐seven studies associated psychological challenges with IMDs [23, 24, 25, 26, 27, 28, 29, 30, 31, 33, 34, 35, 36, 37, 38, 39, 40, 41, 42, 43, 44, 45, 46, 47, 48, 49, 50]. Thirteen of these studies included only parents [26, 27, 29, 30, 35, 36, 38, 40, 41, 42, 43, 45, 47], eight included only patients [25, 31, 34, 37, 39, 48, 49, 50] and six included both groups [23, 24, 28, 33, 44, 46]. These findings were mirrored in lower psychological quality‐of‐life scores than normative data on parent proxy standardised assessment [31, 34, 46]. The impact of IMDs on children's learning and cognition were suggested in eight studies, as parents recounted lack of concentration, ‘slow’ thinking [30], distractibility [49], developmental delay [26, 29] and learning problems [36].

Parents and young people both felt that their IMD affected school performance [24]. Impacted school performance was quantified by lower school functioning scores [38] and low scores across a variety of assessments of executive function [48]. Behavioural issues were noted by parents and young people in seven studies [26, 27, 29, 30, 35, 39, 49]. Some children were described as presenting with defiant behaviour [29], aggression [26, 30, 49] and destructiveness [49]. Young people acknowledged their defiant behaviour, explaining that frustration in their parents' attempts to control their diet fostered a desire to transgress [39]. Behavioural challenges were equally reflected in elevated scores on child behaviour assessments [34].

Parents across 13 studies repeatedly reported the impact of their child's condition on their own mood [24, 27, 30, 33, 34, 36, 38, 41, 42, 43, 44, 45, 47]. Parents highlighted feelings of stress [34, 36, 41, 43, 44], depression [42, 45] and anxiety [24, 30, 33, 38] relating to raising a child with an IMD. The responsibility of caregiving weighed heavily on parents [47], particularly when striving to create a ‘normal’ life for their family [30]. Parents often reported fear and guilt associated with their child's nonadherence [27, 30, 33, 36]. Parents, too, highlighted fear of their child feeling different [24, 33] and fear of what would happen to their child if their caregiver died [33].

Young people reported low self‐esteem [23], feeling embarrassed by their condition [37], feeling different to peers [25] and were worried about their future [24]. Young people tried to avoid feeling different by hiding their diagnosis from peers and struggled to decide with whom they could entrust their diagnosis [39]. Young people noted a ‘profound’ feeling of sadness when offered food that they could not eat and equally feared discrimination and being stigmatised by their differences [25]. A sense of frustration was detailed in three studies, as young people had difficulty with treatments such as limited diets [25, 39] and repeatedly having to explain their condition to others [37].

#### 3.5.4 Practical Challenges

Twelve studies highlighted the practical challenges associated with IMDs [25, 26, 27, 29, 34, 36, 37, 38, 39, 40, 42, 43]. Eight of these studies included only parents [26, 27, 29, 36, 38, 40, 42, 43] and four included only patients [25, 34, 37, 39]. Access to information was noted as a challenge in two studies as parents [40] and young people [39] perceived a dearth of available information surrounding IMDs. Parents noted a general lack of information and understanding of IMDs [40], while young people reported that available information was conflicting [39]. This resulted in families filling their own knowledge gaps. Both significant financial [38, 42] and time burdens [26] were also reported by parents, as were the multitude of tasks required to be completed as a caregiver [29, 36]. Parents in one study associated the greatest volume of stress with the question: How many times did you find yourself thinking about the tasks that you have to do in the last month? [43]. Young people highlighted difficulties tolerating unpalatable food [25], as well as storing medication and interpretation of food labels [37]. Impairment in children's daily activities was reflected in parental reports of increased family impact [38] and low scores on assessments of adaptive function [34] Table 3.

**TABLE 3:** Summary of quantitative findings.

Challenges	Findings	First author, year
Physical		
Scores	Young people with IMDs self‐reported lower physical quality‐of‐life scores on the KINDL than normative data. Siblings self‐reported quality‐of‐life scores that were in the same range as that of healthy children and slightly higher quality‐of‐life scores than their sibling with an IMD	Yamaguchi, 2018 [28]
	IMD parents proxy‐reported significantly lower scores on PedsQL than parents of healthy children	Splinter, 2015 [38]
	IMD parents proxy‐reported lower adaptive function on VABS than normative data	Needham, 2014 [33]
	IMD parents self‐reported significantly lower quality of life on WHOQOL‐BREF than normative data	Fabre, 2013 [32]
	Parents and young people with IMDs reported physical dysfunction on HS‐FOCUS and CHAQ	Raluy‐Callado, 2013 [23]
	Young people with IMDs self‐reported lower scores on the physical domain of CHQ than normative data	Cotugno, 2011 [31]
	Young people with IMDs self‐reported significantly lower physical health scores on PedsQL than healthy controls. IMD parents proxy‐reported significantly lower physical health on PedsQL than parents of healthy children	Storch, 2008 [34]
Social		
Scores	IMD parents self‐reported lower resilience scores on the RSA and lower social support scores on the MSPSS than normative data	Ambler, 2018 [35]
	IMD parents proxy‐reported lower scores on relations with friends and leisure activities dimensions on VSP‐A than normative data	Fabre, 2013 [32]
	Young people with IMDs self‐reported significantly lower psychosocial health and social functioning scores on PedsQL than healthy controls. IMD parents proxy‐reported significantly lower psychosocial health and social functioning scores on PedsQL than parents of healthy children	Storch, 2008 [34]
Psychological		
Scores	IMD parents self‐reported elevated perceived stress scores on PSS	Irannejad, 2018 [43]
	Young people with IMDs self‐reported lower overall quality‐of‐life score on the KINDL than normative data	Yamaguchi, 2018 [28]
	IMD parents self‐reported significantly low GHQ‐12 scores (indicating clinically significant anxiety and depression)	Medford, 2017 [45]
	Parents and young people with IMDs reported lower scores on all domains of the PedsQL than healthy controls	Needham, 2015 [46]
	IMD parents self‐reported significantly higher depression scores on the BDI and anxiety scores on the STAI than parents of healthy children	Gunduz, 2015 [42]
	Young people with IMDs were found to be developmentally behind their age group on the DST‐II	Eminoglu, 2013 [50]
	Family stress and perceived social support most significant predictors of poor quality‐of‐life scores	Fidika, 2013 [41]
	Parents and young people with IMDs reported very low self‐esteem scores (indicating a moderate impact) on the CHAQ	Raluy‐Callado, 2013 [23]
	Young people with IMDs self‐reported significantly lower on the psychological domain of CHQ than the Italian population	Cotugno, 2011 [31]

	IMD parents proxy‐reported significantly elevated scores for destruction, depression and aggression on CBCL	Wu, 2011 [49]
	IMD parents who adjusted their occupation since their child's diagnosis self‐reported lower mental quality of life on TNO‐AZL than parents who had not	Ten Hoedt, 2011 [47]
IMD parents proxy‐reported significantly lower on the ABS‐S2 Independent Functioning subscale and significantly higher on CBCL than healthy controls. IMD parents proxy‐reported significantly lower emotional functioning and school functioning scores on PedsQL than healthy controls. IMD parents self‐reported higher PIP and BSI (indicating more stress and distress) scores than parents of healthy children	Storch, 2008 [34]
Practical		
Finance	Parents proxy‐reported significantly lower scores on the ABS‐S2 Economic subscale than parents of healthy children	Storch, 2008 [34]

Abbreviations: ABS‐S2, adaptive behaviourr scale‐school: second edition; BDI, beck depression inventory; BSI, brief symptom inventory; CBCL, child behaviour checklist: CBCL; CHAQ, childhood health assessment questionnaire; CHQ, child health questionnaire; DST‐II, denver developmental screening test‐II; GHQ‐12, general health questionnaire‐12; HS‐FOCUS, hunter syndrome‐functional outcomes for clinical understanding scale; KINDL, kindl health‐related quality of life; MSPSS, multidimensional scale of perceived social support; PedsQL, pediatricpaediatric quality‐ of‐ life inventory; PIP, pediatricpaediatric inventory for parents; PSS, perceived stress scale; RSA, resilience scale for adults; STAI, state–trait anxiety inventory; TNO‐AZL, questionnaire for adult's health‐related quality of life; VABS, vineland adaptive behaviourr scales; VSP‐A, vécu et santé perçue de l'’adolescent et de l'’enfant; WHOQOL‐BREF, world health organization quality‐of‐life brief scale.

## 4 Discussion

The present review is the first, to the authors' knowledge, to examine the psychosocial impact of IMDs on young people with IMDs and their families. In line with literature concerning the impact of chronic illness on families [51], all of the studies included in this review identified significant practical, social, psychological and/or physical challenges with managing a life‐long condition. Here, experiences were quantified using standardised assessments of physical, psychological and social function and/or quality of life, with many families of children with IMDs reporting scores that were significantly lower than normative data [31, 34, 35, 38].

The psychological challenges noted included studies related to difficulties with behaviour, emotions and/or learning. Learning difficulties included poor concentration [30], distractibility [49] and developmental delay [26, 29], affecting school performance [24, 38]. These findings are in line with the relevant literature, given that the impact of IMDs on learning and development has been well‐documented. Severe intellectual disability has been described as inherent to untreated IMDs due to the build‐up of neurotoxic substances in the brain [52]. Mirroring the experiences outlined in the present review, recent research has reported increased inattention and hyperactivity in children with PKU [53], poor executive function in children with TT1 [54] and slow processing speed in those with MMA [55]. In these studies, clinical presentations were associated with high concentrations or fluctuations in volume of toxic substances in participants' blood. More generally, however, learning outcomes are not thought to be influenced by metabolic control alone but by a network of metabolic and environmental factors [52].

Studies exploring social and emotional challenges highlighted the extent to which children and their parents feared diagnosis‐related discrimination and stigmatisation [25], with both parties particularly concerned with feeling different from others [24, 33, 39]:

I'm ashamed… I feel different. Well, I know I'm not different, but I feel it. I feel it [25].

Young people reported attempting to conceal their diagnosis [39] and feeling embarrassed by their condition [37], which may be consistent with their reports of low self‐esteem [23]. Concerns surrounding feeling different from peers are common among children with chronic illnesses [56], as they often yearn for a ‘normal’ life [57]. Any deviance from group norms can result in social exclusion [58], resulting in low mood and low self‐esteem in those excluded [59]. Young people who require special diets may be particularly vulnerable to such ostracisation, and in turn mental health difficulties, due to the centrality of food at social gatherings such as birthday parties:

If you're talking to a professional, it wouldn't be that embarrassing. But like, if you're telling it to your mates who don't know… [37]

The burden of caring for children with an IMD was clear from the present review, with parents reporting having ‘no social life’ [33]. Parents reported significant stress [34, 36, 41, 43, 44] and anxiety [24, 30, 33, 38], often associated with the heavy weight of their myriad responsibilities [29, 36, 43, 47].

That's just how I've dealt with it…I've taken control…I'm sort of in charge [40].

In line with the findings of the present review, research on caring for children with chronic illness demonstrates how parents are prone to poor physical and emotional health due to overly demanding responsibilities, hectic schedules and poor social support [60]. Interestingly, parents in one of the studies reviewed here distanced themselves from family and friends due to their lack of understanding of IMDs [29]:

They don't get it [37].

It should be noted that while the scope of present review was to examine the challenges faced by families with IMDs, this does not imply that families of children with IMDs experience only challenges. Families from included studies recognised that the diagnosis gave rise to a better perspective on life and stronger family bonds [38]. Equally, not all families reported experiencing a high level of distress [44] and some reported having good support networks [33]. Still, the challenges collated here give rise to implications for clinical practice discussed hereafter.

### 4.1 Clinical Implications

This review provides insights that may be utilised by healthcare providers to develop supports that meet the psychosocial needs common to children with IMDs and their families. Experiencing social exclusion and isolation as a teenager has been associated with low mood and poor self‐esteem, as well as dysregulation and increased retaliatory behaviour [59]. While social exclusion can have a significant negative effect on individuals of all ages, research has suggested that young people may experience increased sensitivity to ostracism relative to their older counterparts [61]. Communication with peers, particularly online, has proven effective in promoting the development of young people's identity and sense of belonging [62]. Online peer support groups may therefore offer young people with the opportunity to connect with those who understand the realities of living with a metabolic condition, fostering a sense of social unity and combatting isolation [58].

Support groups may also be beneficial for parents, providing a space to learn from the experiences of and to find support in other parents of children with IMDs [63]. As mentioned, parents in one of the included studies distanced themselves from family and friends due to their lack of understanding of IMDs [29]. It may therefore also be helpful to provide psychoeducation in the clinic to the wider family network to ensure all stakeholders understand the child's metabolic condition and treatment plan, as well as the implications of nonadherence. The current research group carried out a scoping review of research concerning psychosocial group interventions for parents and/or patients with IMDs, from which only a single study was found [64]. This dearth of literature gives rise to uncertainty surrounding the suitability of psychosocial group interventions within this population and highlights the need for clinicians to evaluate, and in turn disseminate their findings concerning, the effectiveness of group interventions when delivered to families with IMDs.

An understanding of the attention and learning difficulties experienced by children with IMDs may be of use to school staff so that appropriate expectations of young people may be established, as well as appropriate supports arranged. Offering psychoeducation to schools on the clinical manifestations of high blood‐Phe levels in children with poorly controlled PKU, for example, may be helpful in guiding schools towards appropriate in‐class supports and away from misplaced queries of attention‐deficit/hyperactivity disorder (ADHD). While there can be an increased risk of ADHD in IMDs [65], it is important that school staff understand the impact of high phe levels in the short and long term. While clinicians must ensure that families fully understand the importance of adhering to dietary restrictions, it is equally important to recognise the other factors that contribute to the development of learning difficulties, and that children with well‐controlled IMDs can still experiences such difficulties.

Adverse findings from the discussed studies reiterate the need for psychologists and medical social workers to be established as permanent members of the multidisciplinary teams caring for young people with IMDs, to reduce the impact of these findings long term. Failure to recognise the importance of psychology and social work in addressing the needs of young people with IMDs and their families may result in exacerbation of the mentioned psychological, social, physical and practical difficulties throughout the patients' childhood and long into their adulthood.

### 4.2 Strengths and Limitations

To our knowledge, this is the first review to collate research exploring the psychosocial impact of IMDs from both patient and parent perspectives. Both quantitative and qualitative studies have been included in order to provide insight into the experience of IMDs while corroborating these insights with results from standardised assessments of the same. Although a synthesis of such quantitative studies could not be conducted due to heterogeneity between the measures adopted to examine psychosocial impact across studies, [66] the high appraisal scores awarded to the majority of included articles indicate that this review provides a comprehensive synthesis of valid and reliable research. Furthermore, this review bolsters the validity of parental proxies by cross‐referencing findings with those from studies of patient populations.

It should be noted that this review did not specifically account for the potential confounder of co‐occurring diagnoses. Though none of the included studies noted the presence of comorbidities amongst samples, studies did not specify that comorbidities were not present. This renders their likelihood, and in turn their contribution to the psychosocial challenges reported here, difficult to discern. This possibility should be taken into account when interpreting the presented findings.

It should also be noted that the majority of studies included samples of families with PKU only (*n* = 16). As would be expected, challenges were more pronounced for families of children with IMDs that were life‐limiting [33, 46]. Still, the present review highlights the impact of IMDs on all families carrying any diagnosis.

## 5 Conclusion

The present review is the first, to the authors' knowledge, to systematically review the psychosocial challenges faced by children with IMDs and their families. The studies presented here indicate that both parents and young people experience significant physical, social, psychological and practical challenges, irrespective of which metabolic condition they have been diagnosed with. Social isolation, burden of care, and learning and emotional difficulties were among some of the most common experiences reported by young people and their parents. These findings highlight a number of implications for clinical practice including the establishment of peer support groups, the inclusion of psychology and social work in metabolic MDTs, and increased psychoeducation for families and school staff concerning IMDs and their cognitive sequelae, to improve families' experiences and outcomes. This review collated the experiences common to all children with IMDs and their families so that effective trans‐diagnostic supports, which meet the needs of families with various IMDs, may be developed in the future.

## Conflicts of Interest

The authors declare no conflicts of interest.

## Supporting information


Tables S1–S4.

## Data Availability

The authors confirm that the data suporting the findings of this study are available within the article and its Supporting Information.
