# Supplementary material for: Psychosocial Challenges Facing Young People With Inherited Metabolic Disorders and Their Parents: A Systematic Review
Source: JIMD Rep. 2025 Feb 25;66(2):e70000. doi: 10.1002/jmd2.70000 (PMC11860279; doi:10.1002/jmd2.70000)
Supplement: Supplementary file 1 — Tables S1–S4. [file JMD2-66-e70000-s001.docx]

| **Table S1.** Quantitative measures used in included studies | |
| --- | --- |
| **First author, year** | **Measure(s)** |
| Arpaci, 2019 | Caregiver Burden Inventory  Data Collection Form Related to Symptoms and Problems |
| Morawska, 2019 | PKU Impact and Treatment Quality of Life Questionnaire  Family Background Questionnaire  Parenting Stress Index (PSI)—Short Form  Child Adjustment and Parent Efficacy Scale  30-item Parenting Scale |
| Ambler, 2018 | General Health Questionnaire  Resilience Scale for Adults  Multidimensional Scale of Perceived Social Support  Eyberg Child Behavior Inventory  Developmental Behaviour Checklist Parent/Carer Version  Bespoke child dependency questionnaire |
| Irannejad, 2018 | Perceived stress scale  SF36 quality of life questionnaire |
| Yamaguchi, 2018 | Japanese version of KINDL  World Health Organization QoL BREF  Parenting Stress Index–Short Form  Family Empowerment Scale |
| Campbell, 2017 | TYR-QOL |
| Medford, 2017 | General Health Questionnaire-12  Pediatric Inventory for Parents  Resilience Scale for Adults  Scale of Perceived Social Support |
| Witalis, 2016 | Bespoke questionnaire |
| Gunduz, 2015 | State-Trait Anxiety Inventory  Beck Depression Inventory |
| Needham, 2015 | Pediatric Quality of Life Inventory |
| Splinter, 2015 | Pediatric Quality of Life Inventory |
| Needham, 2014 | Vineland Adaptive Behavior Scales  PedsQL Family Impact Module |
| Eminoglu, 2013 | KINDL questionnaire  QoL Scale for Metabolic Diseases–Parent Form  Kovacs Children’s Depression Inventory Parent Form  Denver Developmental Screening Test–II |
| Fabre, 2013 | Vécu et Santé Perçue de l’Adolescent et de l’Enfant  WHOQOL-BREF |
| Fidika, 2013 | Impact on Family Scale  The Coping Health Inventory for Parents  Social Support Questionnaire- Short Form 22  The Ulm Quality of Life Inventory for Parents |
| Raluy-Callado, 2013 | Hunter Syndrome-Functional Outcomes for Clinical Understanding Scale  Childhood Health Assessment Questionnaire  Child Health Questionnaire  Health Utilities Index |
| Cotugno, 2011 | Child Health Questionnaire |
| ten Hoedt, 2011 | TNO-AZL Questionnaire for Adult’s HRQOL  Bespoke questionnaire |
| Wu, 2011 | Bayley Scales of Infant Development  Peabody Developmental Motor Scale II  Toddler Temperament Questionnaire  Child Behavior Checklist |
| Hatzmann, 2009 | TNO-AZL Questionnaire for Adult’s Health-related Quality of life |
| Lord, 2008 | Malaise Inventory  Child Behavior Checklist  Hunter Opinions and Personal Expectations  Ways of Coping Questionnaire |
| Storch, 2008 | Pediatric Quality of Life Inventory  Asher Loneliness Scale  Child Behavior Checklist  Brief Symptom Inventory  Pediatric Inventory for Parents  McMaster Family Assessment Device  AAMR Adaptive Behavior Scale-School: Second Edition  Sibling Relationship Questionnaire |
| Van Zutphen, 2007 | Delis–Kaplan EF Battery |
| Bilginsoy, 2005 | Bespoke questionnaire  Wechsler Abbreviated Scales of Intelligence |

| **Table S2.** Critical appraisal of cross-sectional studies cont. | | | | | | | |
| --- | --- | --- | --- | --- | --- | --- | --- |
| Author, Year | Raluy-Callado, 13 | Splinter, 16 | VanZutphen, 18 | Ten Hoedt, 11 | Witalis, 16 | Wu, 11 | Yamaguchi, 18 |
| Were the criteria for inclusion in the sample clearly defined? |  |  |  |  |  |  |  |
| Were the study subjects and the setting described in detail? |  |  |  |  |  |  |  |
| Was the exposure measured in a valid and reliable way? | n/a | n/a | n/a | n/a | n/a | n/a | n/a |
| Were objective, standard criteria used for measurement of the condition? |  |  |  |  |  |  |  |
| Were confounding factors identified? |  |  |  |  |  |  |  |
| Were strategies to deal with confounding factors stated? |  |  |  |  |  |  |  |
| Were the outcomes measured in a valid and reliable way? |  |  |  |  |  |  |  |
| Was appropriate statistical analysis used? |  |  |  |  |  |  |  |
| **Total (/7)** | **5** | **4.5** | **4** | **7** | **4** | **5** | **6.5** |
| **Note.** Denotes a clear finding Denotes unclear finding | | | | | | | |

| **Table S3.** Critical appraisal of case-control studies | |
| --- | --- |
| Author, Year | Storch, 21 |
| Were the groups comparable other than the presence of disease in cases or the absence of disease in controls? |  |
| Were cases and controls matched appropriately? |  |
| Were the same criteria used for identification of cases and controls? |  |
| Was exposure measured in a standard, valid and reliable way? | n/a |
| Was exposure measured in the same way for cases and controls? | n/a |
| Were confounding factors identified? |  |
| Were strategies to deal with confounding factors stated? |  |
| Were outcomes assessed in a standard, valid and reliable way for cases and controls? |  |
| Was the exposure period of interest long enough to be meaningful? | n/a |
| Was appropriate statistical analysis used? |  |
| **Total (/7)** | **5.5** |
| **Note.** Denotes a clear finding Denotes unclear finding | |

| **Table S4.** Critical appraisal of qualitative studies | | | | | |
| --- | --- | --- | --- | --- | --- |
| Author, Year | Bose, 21 | Carpenter, 18 | Di Ciommo 12 | Sharman, 13 | Vegni, 09 |
| Is there congruity between the stated philosophical perspective and the research methodology? |  |  |  |  |  |
| Is there congruity between the research methodology and the research question or objectives? |  |  |  |  |  |
| Is there congruity between the research methodology and the methods used to collect data? |  |  |  |  |  |
| Is there congruity between the research methodology and the representation and analysis of data? |  |  |  |  |  |
| Is there congruity between the research methodology and the interpretation of results? |  |  |  |  |  |
| Is there a statement locating the researcher culturally or theoretically? |  |  |  |  |  |
| Is the influence of the researcher on the research, and vice- versa, addressed? |  |  |  |  |  |
| Are participants, and their voices, adequately represented? |  |  |  |  |  |
| Is the research ethical according to current criteria or, for recent studies, and is there evidence of ethical approval by an appropriate body? |  |  |  |  |  |
| Do the conclusions drawn in the research report flow from the analysis, or interpretation, of the data? |  |  |  |  |  |
| **Total (/10)** | **8** | **9** | **5.5** | **9** | **7.5** |
| **Note.** Denotes a clear finding Denotes unclear finding | | | | | |
